# Supplementary material for: Identification and functional analysis of non-coding regulatory small RNA FenSr3 in Bacillus amyloliquefaciens LPB-18
Source: PeerJ. 2023 May 15;11:e15236. doi: 10.7717/peerj.15236 (PMC10194069; doi:10.7717/peerj.15236)
Supplement: Supplemental Information 4 [file peerj-11-15236-s004.zip › KO/CK-vs-T1_map/map04915.html]

KEGG PATHWAY: Estrogen signaling pathway - Reference pathway


|  |  |
| --- | --- |
| **Estrogen signaling pathway - Reference pathway** |  |

[
Pathway menu
| Organism menu
| Pathway entry
| Show description
| User data mapping
]

Estrogens are steroid hormones that regulate a plethora of physiological processes in mammals, including reproduction, cardiovascular protection, bone integrity, cellular homeostasis, and behavior. Estrogen mediates its cellular actions through two signaling pathways classified as "nuclear-initiated steroid signaling" and "membrane-initiated steroid signaling". In the "nuclear" pathway, estrogen binds either ERalpha or ERbeta, which in turn translocates to the nucleus, binds DNA at ERE elements and activates the expression of ERE-dependent genes. In "membrane" pathway, Estrogen can exert its actions through a subpopulation of ER at the plasma membrane (mER) or novel G-protein coupled E2 receptors (GPER). Upon activation of these receptors various signaling pathways (i.e. Ca2+, cAMP, protein kinase cascades) are rapidly activated and ultimately influence downstream transcription factors.

|  |  |  |
| --- | --- | --- |
| Reference pathway | 184% 150% 122% 100% 82% 67% 55% | 图片下载 |
